# Targeted analysis of Ubiquitin-Specific Peptidase (USP8) in a population of Iranian people with Cushing’s disease and a systematic review of the literature

**DOI:** 10.1186/s12902-024-01619-z

**Published:** 2024-06-11

**Authors:** Nahid Hashemi-Madani, Sara Cheraghi, Zahra Emami, Ali Zare Mehrjardi, Mahmoud Reza Kaynama, Mohammad E. Khamseh

**Affiliations:** 1 Endocrine Research Center, Institute of Endocrinology and Metabolism, Iran University of Medical Sciences, Tehran, Iran, No. 10, Firoozeh St., Vali-asr Ave., Vali-asr Sq, Tehran, Iran; 2 Department of Pathology, Firoozgar hospital, Iran University of Medical Sciences, Tehran, Iran; 3 Department of Endocrinology, Arad Hospital, Tehran, Iran

**Keywords:** Ubiquitin-specific peptidase 8 (USP8), Ubiquitin-specific peptidase 48 (USP 48), Cushing’s disease, Mutation, Corticotroph adenoma

## Abstract

**Objective:**

Activating mutation in Ubiquitin-specific peptidase (*USP8*) is identified to enhance cell proliferation and adrenocorticotropic hormone (ACTH) secretion from corticotroph pituitary adenoma. We investigated the *USP8* variant status in a population of Iranian people with functional corticotroph pituitary adenoma (FCPA). Moreover, a systematic review was conducted to thoroughly explore the role of *USP8* variants and the related pathways in corticotroph adenomas, genotype-phenotype correlation in *USP8*-mutated individuals with FCPA, and the potential role of *USP8* and epidermal growth factor receptor (EGFR) as targeted therapies in PFCAs.

**Methods:**

Genetic analysis of 20 tissue samples from 19 patients with PFCAs was performed using Sanger sequencing. Moreover, a systematic literature review was performed using the Preferred Reporting Items for Systematic Reviews and Meta-Analyses (PRISMA) guidelines. PubMed, Scopus, web of Sciences, and Cochrane databases were searched. The last search was performed on 20 September 2023 for all databases.

**Results:**

In our series, we found two somatic mutations including a 7-bp deletion variant: c.2151_2157delCTCCTCC, p. Ser718GlnfsTer3, and a missense variant: c.2159 C > G, p. Pro720Arg (rs672601311) in exon 14. The Systematic review indicated *USP8* variant in 35% of corticotroph adenomas, with the highest frequency (25%) in 720 code regions, p. Pro720Arg. Data regarding the impact of *USP8* mutational status on clinical characteristics and outcomes in FCPAs are inconsistent. Moreover, Pasireotide as well as inhibitors of EGFR such as Gefitinib and Lapatinib, as well as *USP8* inhibitors including -ehtyloxyimino9H-indeno (1, 2-b) pyrazine-2, 3-dicarbonitrile, DUBs-IN-2, and RA-9 indicated promising results in treatment of corticotroph adenomas.

**Conclusion:**

Although the USP8*-*EGFR system has been identified as the main trigger and target of corticotroph tumorigenesis, more precise multicenter studies are required to yield more consistent information regarding the phenotype-genotype correlation and to develop effective targeted therapies.

**Supplementary Information:**

The online version contains supplementary material available at 10.1186/s12902-024-01619-z.

## Introduction

Pituitary corticotroph adenoma accounts for 68% of endogenous hypercortisolism [1]. Prolonged exposure to high cortisol levels is associated with a variety of long-term complications, impaired quality of life, and increased mortality [2]. Transsphenoidal surgical excision is the treatment of choice. However, curative surgery is challenging with the initial remission rate of 65–85% and a high recurrence rate [3, 4].

The majority of functional corticotroph adenomas (FCAs) are sporadic. Although the genetic background is not well-established, potential candidate genes are proposed for tumor initiation and progression [5]. Hotspot mutations in ubiquitin-specific peptidase (*USP8*) are reported in 11–62% of sporadic corticotroph adenomas [6–8]. USP8 is a deubiquitinating enzyme that plays an important role in enhancing cell proliferation and regulating cell cycle [9]. The mutant *USP8* was found to activate the epidermal growth factor receptor (EGFR) signaling pathway ultimately promoting adrenocorticotrophic hormone (ACTH) secretion [6]. Moreover, overexpression of EGFR and its signaling pathway components in pituitary corticotroph adenoma was reported [10]; and found to be positively associated with ACTH and cortisol levels as well as tumor recurrence [10]. These outcomes suggest that USP8 and EGFR are promising biomarkers for prediction of recurrence and can be used as targeted therapy.

Thus, we conducted a study to examine the *USP8* and ubiquitin-specific peptidase 48 *(USP48*) variations in a group of Iranian people with Cushing’s disease (CD) and carried out a systematic review of the literature regarding the USP8/EGFR and their potential role in the clinical outcomes and targeted therapy in CD.

## Methods

### Case series

#### Study population

Paraffin-embedded blocks of pituitary tumor tissue from 19 patients with ACTH-secreting pituitary adenoma who underwent transsphenoidal surgery (TSS) between 2011 and 2019 were examined. The diagnosis of CD was based on clinical features and biochemical criteria [11]. The patients clinically suspected to CD were asked to collect urine free cortisol (UFC) in two separated times and underwent overnight dexamethasone suppression test (ODST). After confirmation of ACTH-dependent Cushing’s syndrome using measurement of ACTH level, a high-dose dexamethasone suppression test (HDDST) was performed to confirm the pituitary source of hypercortisolism. Patients with equivocal results or those with pituitary tumors less than 6 mm in size were undergone inferior petrosal sinus sampling (IPSS). Patient with clinical, biochemical, and radiological evidences of CD were undergone TSS. And eventually, corticotroph adenoma was confirmed using immunohistochemically staining of tumor tissue in all patients. The study was approved by the IUMS Research Ethics Committee (IR.IUMS.REC.1398.082). It was carried out under the declaration of Helsinki and the International Conference on Harmonization of Good Clinical Practice (ICH-GCP) guidelines, and informed consent was obtained from all patients.

#### DNA extraction and Sanger sequencing

A 10-µm thick section of formalin-fixed and paraffin-embedded (FFPE) tissue per sample was used for genomic DNA extraction. A molecular test was performed by amplification of *USP8* and *USP48* hotspot exons (exon 14 and exon 10, respectively) using conventional polymerase chain reaction (PCR). *USP8* was amplified by two primer pairs; USP8_F1: AGCAGAATACTTTGGAGTGATTTC and USP8_R1: TTTGGAAGGTTCCCTATCCC with 251 bp product, USP8_F2: ACCCCTCCAACTCATAAAGC and USP8_R2: GAGTAGAAACTTTGAAATACAGCAC, with 220 bp product. A 240 bp fragment of *USP48* was produced using; USP48_F: CCCGCTAAAGAATAAACAAACTC and USP48_R: GCATTCTAAAACATTTGCCTGC. PCR was done in 25 µl final volume (Ampliqon 2x PCR Mix) containing 0.5 µM of each primer and 30 ng of genomic DNA for 35 cycles (94 °C for 20 s, annealing 60 °C for 20 s and extension 72 °C for 20 s). The quality of PCR products was assessed by 2% agarose gel electrophoresis. Bidirectional Sanger sequencing was performed on an ABI DNA Analyzer (Applied Biosystems), The PCR primers were also used in the sequencing reaction. CodonCode Aligner software was used to analyze hotspot exome sequencing. Sequencing data quality was evaluated using Sanger electropherograms of both forward and reverse strands. The identified somatic mutations were analyzed in DNA taken from whole blood samples, but germline mutation was not detected.

### Systematic review

#### Overview of the systematic literature review

We performed a systematic review of the literature to identify all published papers that reported the frequency of the *USP8* variant and the related pathways in corticotroph pituitary adenomas, detailed clinical presentation and outcomes of patients with and without USP8 mutation and examined the USP8 and EGFR as targeted therapy.

#### Search strategy

We searched the PubMed, Scopus, web of Sciences, and Cochrane databases. The date of the last search was 20 September 2023 for all databases. We did not apply any language restrictions. Search terms included: “Cushing disease”, “Cushing’s disease”, “Corticotroph adenoma”, “Cushing adenoma”, “Client Cushing disease”, “Atypical corticotroph tumor”, “Corticotroph carcinoma”, “Normal pituitary”, “Corticotroph adenoma”, “Corticotroph Tumor”, “Pituitary ACTH Hypersecretion”, “ACTH-Secreting Pituitary Adenoma”, “Mutation”, “Germline mutation”, “Sporadic mutation”, USP8, “ubiquitin specific peptidase 8”, USP48, “ubiquitin specific peptidase 48”, “Epidermal growth factor”, EGF, “Epidermal growth factor receptor” EGFR, Biomarker.

#### Inclusion and exclusion criteria

All published papers including original articles, case reports, and case series were included in this systematic review provided that they have reported the frequency of *USP8* variant or EGFR expression in corticotroph pituitary adenomas, compared the clinical presentation and outcomes of patients with and without *USP8* variant, or examined USP8 or EGFR as treatment targets in CD. Studies applying any type of tissue namely resected human pituitary adenoma tissue, primary cell cultures, cell lines, and transfected cells were included. Articles were excluded if they included different types of pituitary tumors and did not separately analyze corticotroph adenomas, or if they were written in any language other than English.

## Results

### Case series

#### Baseline characteristics of the participants

This study included 19 patients of whom 63% (*n* = 12) were women. They aged between 17 and 65 years. Baseline cortisol ranged between 20 and 43 mic/dl. The ACTH level ranged between 34 and 164 pg/ml. The basal UFC ranged between 316 and 1153 mic/24 h. All patients presented with micro-adenoma except for two patients, one man and one woman (supplementary Table 1).

#### Frequency of USP8 gene variants

**Fig. 1:**
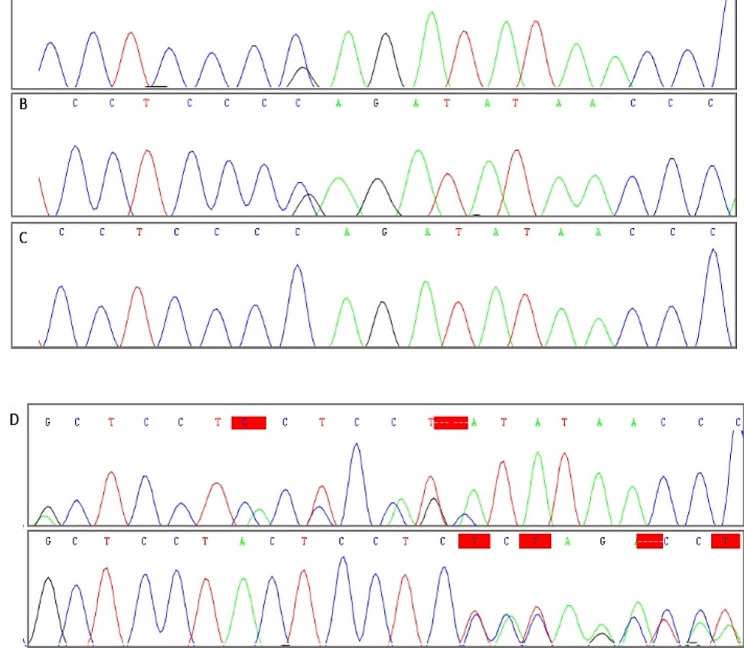
Sanger sequencing of pathogenic variants in USP8 hotspot exon. **(A, B)** bi-directional sequencing of heterozygous missense variant, c.2159 C > G, in tissue sample, **(C)** A Sanger sequencing chromatogram of the blood sample detected no germline c.2159 C > G mutation. **(D)** Sanger sequencing chromatograms confirm the presence of heterozygous deletion (c.2151_2157delCTCCTCC) in tissue sample of patient II

Sanger sequencing of 20 CD tumors revealed two heterozygous pathogenic variants in 2 samples: the 7-bp deletion variant, c.2151_2157delCTCCTCC, p. Ser718GlnfsTer3 was found in one patient; another patient showed the missense variant, c.2159 C > G, p. Pro720Arg (rs672601311) in exon 14. The pathogenic variants were found only in tumor tissue. Targeted sequencing (exon 10) of *USP48* did not detect any pathogenic variant. The somatic variations in our study are in the catalytic conserved domain of USP8 protein and lead to disruption of the interaction between USP8 catalytic domain and 14-3-3 protein (Fig. 1).

#### Clinical outcomes after surgery

All patients achieved biochemical and structural cures after surgery except for one man and one woman who suffered from persistent disease because the tumors were not completely resected due to invasion into the cavernous sinus. They underwent radiotherapy after surgery. These two patients did not show the *USP8* variant. Moreover, one man without evidence of the *USP8* variant and the two women with the *USP8* variant presented with recurrence after initial remission. They presented with micro-adenoma before surgery (supplementary Table 1).

### Systematic review

**Fig. 2:**
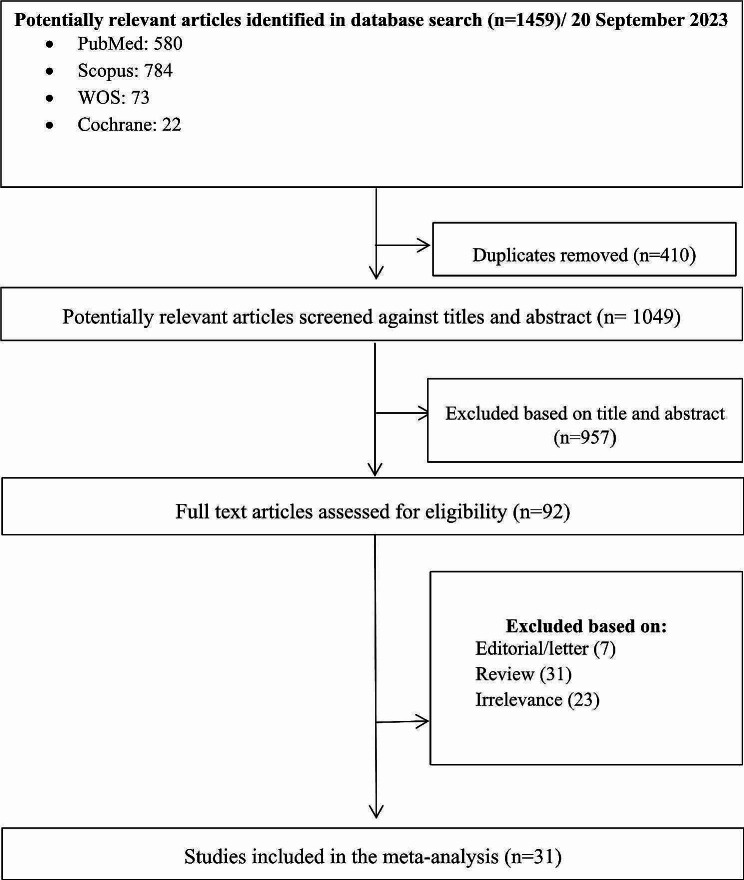
Flow diagram of literature search and study selection

The search yielded 1459 initial results. Upon removing the duplications (*n* = 410), 1049 studies were reviewed based on the relevancy of their titles and abstracts. Having excluded 957 articles, 92 studies were selected for full-text review. After an in-depth review, 31 articles were selected based on the inclusion and exclusion criteria. A PRISMA diagram detailing the search results is shown in Fig. 2.

**Fig. 3:**
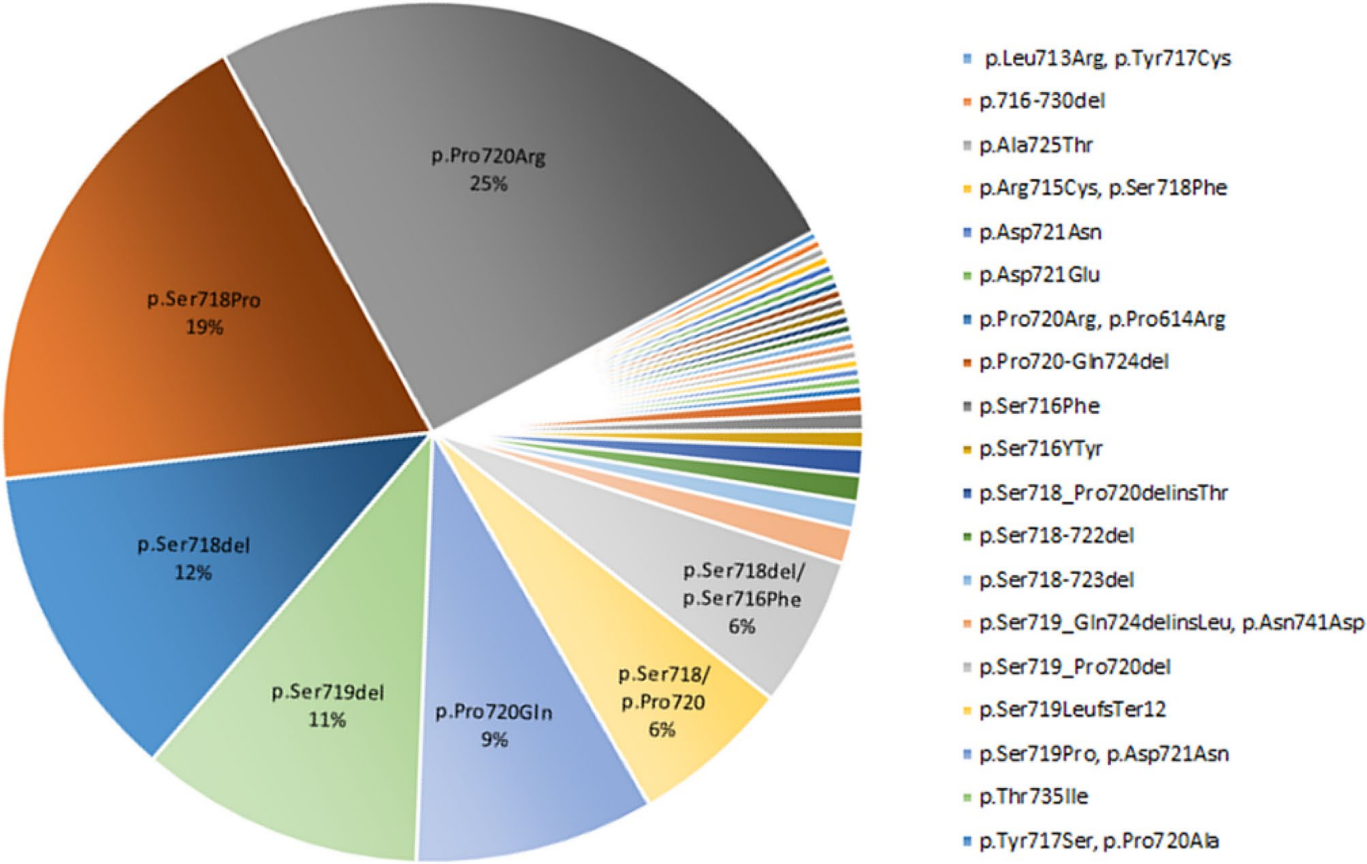
Summary of USP8 mutations in patients with CD in selected studies

In this systematic review we extracted the information regarding the *USP8* variant and the EGFR system in corticotroph adenomas. The *USP8* variant was found in 460 individuals with FCPA accounting for 35% of the population included in the related published series (Table 1). Moreover, the highest frequency of missense mutation was found in the 720 code region, p.Pro720Arg (25%), followed by 19% in p.Ser718Pro (Fig. 3). In addition, the frequency of frame-shift and in-frame deletion observed in p.Ser718del and p.Ser719del was 12% and 11%, respectively (Fig. 3).

**Table 1:** Results of systematic literature review

Author, Year	Study Population (*N*)	Female *N* (%)	Age	Tumor size/invasiveness in USP8-Mut. vs. Wilde-type	Mutations	Patients with the Mut. (*N*)	Recurrence	Remission after surgery	Hormonal Profile Mut. vs. Wild	Macro-Corticotropinomas	Micro-Corticotropinomas	Genes Expression	Expression In Mut.-USP8
Theodoropoulou M et al. 2004 [18]	16			NA	-	-	-	NA	NA	-	-	EGFR	-
												P27/Kip1	Low
Perez-Rivas LG et al. 2015 [22]	134	98(73.1)	33.75 ± 17	No difference	p.S718P, p.S718del, p.P720Q, p.P720R, p.P720_D721del, p.S719_P720del, p.S719_T723del, p.S719_Q724del	48	NA	Low	No difference	65	69	-	-
Reincke M.et al. 2015 [6]	17	11(64.7)	18–56	Lower	p.Ser718del, p.Ser718Pro, p.Ser718Cys, 2138T-> G + 2150 A-> G, p.[Leu713Arg; Tyr717Cys], p.Ser718Pro, p.Pro720Arg	6	NA	NA	High	7	10	EGFR, POMC	High
Ma ZY et al. 2015 [7]	120	-	36	low	p.716-730del, p.D721N, p.P720Q, p.P720R, p.A725T, p.PD720-721del, p.PDIT720-723 L, p.R715C, p.S718F, p.S718del, p.S716F, p.S718F, p.S718P, p.S719del, p.S719P, p.D721N, p.T735I, p.Y717S, p.P720A	75	No difference	No difference	High ACTH	-	-	-	-
Hayashi K et al. 2016 [30]	60	47(78.3)	45	Smaller size	p.S718del, p.P720R, p.S719P, p.S718F, p.S718_P720delinsT, p.P720Q, p.P720_D721del	21	NA	High	Low ACTH	32	28	SSTR5, DRD2, MGMT, MSH6, POMC	High
												EGFR, SSTR2	No difference
Faucz FR et al. 2017 [23]	42	27(64.3)	13.7 ± 3.4	No difference	p.S718C, P.S718P, p.S718del, p.S718-723del, p.P720R	13	High	No difference	No difference	-	-	-	-
Albani A et al. 2018 [24]	48	38(79.2)	50 ± 11	NA	p.P720R, p.S718del, p.S718P, p. S719_Q724del, p.P720Q	18	High	No difference	No difference	12	27	-	-
Ballmann C et al. 2018 [12]	42	27(64.3)	42.5 ± 14.4	No difference	p.P720R, p.S716Y, p.S716F, p.S718P, p.P720Q	11	NA	NA	NA	14	26	EGFR	none of the Mut. Were EGFR-positive
Bujko M et al. 2019 [14]	28	25(89.3)	23–76	NA	p.S718del, p.P720R, p.S718P and p.P720Q	11	NA	NA	NA	17	11	POMC, CDC25A, MAPK4	High
												CCND2,CDK6,CDKN1B	Low
Losa M. et al. 2019 [25]	92	73(79.3)	38.6 ± 14	In microadenomas, USP8-mutated tumors had a significantly larger maximum tumor diameter	-	22	No difference	High	No difference	-	-	-	-
Weigand I et al. 2019 [20]	75	53(70.6)	-	NA	NA	17	NA	NA	No difference	-	-	EGFR, CDK, p27/kip1	Low
												HSP90	High
												CREB, TR4	No difference
Liu X et al. 2019 [10]	52	-	35.2 ± 12.4	No correlation	-		High	52	High	-	-	EGFR	-
Castellnou S et al. 2020 [26]	30	24(80)	11–73	No difference	p.S719del, p.P720R, p.S718C, p.S718P, p.P720-Q724del, p.D721E, p.S719_Q724delinsLeu, p.N741D	11	No difference	NA	No difference	12	18	SST5	High
Martins CS et al. 2020 [16]	32	31(96.9)	11–64	High	p.P720R, p.S718del, p.S718P, p.S719_T723del	10	NA	No difference	No difference	8	24	CDKN1B, CCNE1, CCND1, CDK2, CDK4,CDK6	No difference
Sesta A et al.2020 [13]	126	97(77)	40.1	No difference	p.S718, p.P720, p. S719 del, p.P720_723 del	29	NA	NA	High	0.4	0.6	-	-
Bujko M et al. 2021 [28]	26	23(88.5)	49	No difference	-	11	NA	High	No difference	-	-	-	-
Araki T et al. 2021 [19]	32	22(68.8)	16–73	NA	-	6	8	NA	High	20	12	POMC	Higher
Treppiedi D et al. 2021 [27]	60	45(75)	45 ± 15.1	More macroadenoma	p.P720R, p.P720Q, p.S718del	7	High	No difference	No difference	9	39	-	-
Abraham AP et al. 2022 [29]	46	38(82.6)	29.5 ± 8.2	No difference	p.P720R, p.P720Q, p.S718P, p.S719del	17	High	No difference	No difference	17	29	-	-
Albani A et al. 2022 [31]	75		48 ± 13		P.S718del, p.S718P, p.P720R, p.S718P, p.S719LeufsTer12	34	NA	NA		17	34	SSTR5	High
Chen Z et al. 2022 [21]	15	13(86.6)	20–56	NA	p.S718del, p.SSPDL718-722del, p.S718P, P.P720R	10	NA	NA	NA	-	-	ASCL1	No difference
Mossakowska BJ et al. 2022 [17]	47	38(80.9)	20–78	NA	p.P720R, p.P720Q, p.S718SP, p.S719del	4	NA	NA	NA	-	-	CDKN1B, CDK6, CCND2	Low
												CDC25A	High

*The animal model studies were not listed in this table. NA: Not Assign, Mut: Mutated

#### *USP8* variants and the related pathways in corticotroph adenomas

In a study of 42 patients with corticotroph adenomas, *USP8* variants were as follows: p. P720R (found in five patients), p. S718P (found in two patients), p. P720Q (found in two patients), p. S716Y (found in one patient), and p. S716F (found in one patient) [12]. Another genetic study demonstrated mutated *USP8* deubiquitinating EGFR more effectively than wild type *USP8*. Some variants namely p.S718del, p.718SP, and p.P720R have higher deubiquitinated activity, while others including p.S718C, p.L713R, and p.Y717C showed similar activity compared to the wild type. These variants have been shown to increase the catalytic and proteolytic activity of *USP8*, which ultimately leads to the activation of the EGFR pathway. High EGFR levels, in turn, stimulate POMC gene transcription and increase plasma ACTH levels [6]. In the study of Seata, the *USP8* variant was found in 23% of corticotroph adenomas. The variants were heterozygous, including p.S718, p.P720 (*n* = 18), p.S719del (*n* = 10), and p.P720_723 del (*n* = 1). Moreover, a comparison of 5 *USP8* mutant vs. 34 wild-type specimens indicated different gene expression profile. According to the results, 2 genes involving in EGF signaling, CMTM8 (CKLFlike MARVEL transmembrane domain containing 8) and MAPK15 (mitogen-activated protein kinase 15), were upregulated in *USP8* variant carriers [13]. Bujko et al. found *USP8* mutation in 31.3% of patients with FCA and silent corticotroph adenomas (SCA). In-frame and missense mutations were p.Ser718del (7 patients), p.Pro720Arg (5 patients), p.Ser718Pro (2 patients) and p.Pro720Gln (one patient). *USP8*-mutated adenomas showed higher level of POMC, CDC25A, MAPK4 but lower level of CCND2, CDK6, CDKN1B than *USP8*-wild-type tumors [14].

Another study investigated the molecular pathogenesis of the spectrum of corticotroph adenomas, including CD, SCA, CCA (Crooke cell adenomas), and ACTH-producing carcinoma using whole exome sequencing. The patients with ACTH-producing carcinoma showed the highest number of variants in *USP8*, EGFR, TP53, AURKA, CDKN1A, and HSD3B1 genes. The *USP8* variant was found in c.2159 C > G (p.Pro720Arg) and was positively correlated to the tumor size. However, the *USP8* variant was not present in any of the patients with CD [15].

Martins and colleagues conducted a study to investigate the *USP8* variant and its contribution to gene expression of cell cycle regulators including P27/CDKN1B, CCNE1, CCND1, CDK2, CDK4, and CDK6 in 32 corticotroph adenoma. They identified variants in certain hotspot exons, namely p.720R (found in five patients), p.S718del (found in three patients), p.S718P (found in one patient), and p.S719_T723del (found in one patient). Moreover, there was no difference regarding the gene expression of the cell cycle regulators CDKN1B (P27), CCNE1 (CYCLIN-E1), CCND1 (CYCLIN-D1), CDK2, CDK4, and CDK6 according to *USP8* variant status [16]. Another study investigating the *USP8* variants and genes involved in cell cycle regulation observed *USP* variants including p. P720R (*n* = 8), p.720Q (*n* = 2), p. S718SP (*n* = 2), and an in-frame deletion at the 719 position (*n* = 8). However, USP8-mutated tumors showed lower CDKN1B, CDK6, CCND2 and higher CDC25A expression. They also observed a significantly lower level of p27 in *USP8*-mutated tumors as compared to the wild-type ones [17].

A comprehensive study determined the presence of EGFR at the protein and mRNA levels in different pituitary adenomas. The highest incidence of EGFR expression was found in corticotroph adenomas. The corticotroph adenomas with EGFR expression did not show p27 immunoreactivity [18].

DNA methylation regulates promoter activities. The study by Araki et al. identified a novel regulatory region in the human POMC gene which functions as a second promoter. Moreover, they indicated that this region is highly methylated in SCAs and highly demethylated in FCAs and ectopic ACTH-secreting tumors. They also demonstrated demethylation of the second promoter is associated with aggressive features of FCAs independent of the *USP8* variant or EGFR signaling. In contrast, the first promoter was highly demethylated in *USP8*-mutated FCAs [19]. Weigand et al. indicated that p27/kip1 protein expression significantly decreased in *USP8-*mutated adenomas compared to the wild-type *USP8* tumors. Moreover, higher expression of heat shock protein 90 (HSP90) and an increase in the phosphorylation of the transcription factor CREB was observed in mutated-*USP8* adenomas [20]. Achaete-scute complex homolog 1 (ASCL1) plays an important role in cell proliferation and also regulates POMC in the cell line. In a recent study, genetic analysis of corticotroph adenomas using RNA-seq and IHC showed an increase in ASCL1expression and protein levels in both mutated and wild type *USP8* among CD patients [21].

#### Genotype-phenotype correlation in *USP8*-mutated individuals with functional corticotroph adenoma

Sanger sequencing of 120 FCPAs indicated the somatic *USP8* variant more frequently in women than in men, which was associated with a significant lower size and higher ACTH level. Moreover, compared to the wild-type tumors, the *USP8*-mutated ones display a higher level of EGFR expression with a higher staining intensity. The initial remission rate and the recurrence rate in patients initially receiving remission were comparable in both groups [7]. Another study of patients with 134 functional and 11 silent corticotroph adenomas demonstrated somatic *USP8* variants only in functional adenomas, none of them occurred in silent adenomas. The *USP8* variant in adults was associated with lower age, and predominantly occurred in women. Moreover, the presence of *USP8* variant was inversely associated with remission [22]. In a cohort of 42 pediatric patients with FCA, five different *USP8* variants (three missenses, one frame-shift, and one in-frame deletion) were identified. None of the patients were found to have gremlin *USP8* variants. Patients with somatic *USP8* variant were significantly older than those with wild-type *USP8*. However, there was no significant difference in terms of preoperative hormonal profile and tumor invasiveness between the two groups. However, somatic *USP8* mutated patients showed a higher rate of recurrence after a mean follow-up of 34.7 months [23].

In a cohort of 48 FCA, patients with the *USP8* variant had significantly higher levels of preoperative urine-free cortisol (UFC). But there was no difference in preoperative ACTH and cortisol level between *USP8*-mutated and wild-type groups. Although initial remission rate was similar in both groups, patients with *USP8* variant revealed a significantly higher rate of recurrence within 10 years follow-up, with a significantly shorter time to recurrence [24]. *USP8*-mutated FCA patients presented with a significantly larger size of adenoma in a retrospective study. But preoperative hormonal profile and the remission rate were similar in both groups [16]. Retrospective genetic analysis of 92 FCA patients indicated that the *USP8* variant was significantly higher in women than men. There was no significant difference in preoperative hormonal profile and tumor size between *USP8*-mutated and wild-type groups. *USP8*-mutated carriers were more likely to achieve surgical remission. However, after 10 years follow-up, the recurrence rate was similar in the both groups [25]. A Retrospective study of patients with 30 functional and 20 silent corticotroph adenomas showed *USP8* variants in 11 and 2 adenomas, respectively. There was no difference in sex, age, preoperative hormonal profile, and size of the adenomas between patients with and without *USP8* variants. However, the *USP8*-mutated tumors revealed a higher rate of invasiveness. Furthermore, somatostatin receptor 5 (SSRT5) was more frequent in *USP8*-mutated adenomas [26]. In a retrospective study of FCA patients found no difference considering age at the presentation and hormonal profile between patients with and without *USP8* variants. However, macro-adenoma was more frequently seen in USP8-mutated patients. Although initial remission rate was similar in the both groups, after a median 5 (2–8) years of follow-up, *USP8*-mutated carriers were more likely to develop recurrence [27]. The study conducted by Bujko et al., comparing patients with *USP8* mutated and wild-type corticotroph adenomas, demonstrated no difference in age, sex, preoperative hormonal profile, tumor invasiveness, proliferation index, and histology (sparsely vs. densely granulation) between the two groups. However, the USP8-mutated patients showed a higher rate of remission [28].

A cohort of Asian-Indian patients with CD identified that there was no significant difference considering age, sex, tumor size, tumor invasion, and preoperative hormonal profile of the participants with and without the *USP8* variant. Moreover, the initial remission rate and long-term recurrence, after a mean follow-up of 25.3 ± 13.6 months, were also comparable in both groups [29]. Liu et al. investigated the expression of EGFR and its signaling pathways in FCAs. They demonstrated that EGFR was overexpressed in 29 of 52 patients with FCA. Moreover, the EGFR signal transducing molecules p-EGFR, p-Akt and p-Erk were upregulated in EGFR-overexpressing adenomas but not in EGFR-negative adenomas. Moreover, the expression of EGFR was positively correlated with ACTH and cortisol levels but not with age, sex, or adenoma size. After a mean follow-up of 42.8 months, 22 patients had tumor recurrence. The EGFR expression was positively associated with the recurrence rate [10].

#### USP8 and EGFR as potential therapeutic targets in functional corticotroph adenoma

Our systematic search yields nine studies investigating the possible role of the *USP8* variant in response to the medications. Four studies evaluated the presence of SSTR5 receptors in *USP8*- mutated tumors. Genetic analysis of FCAs from a cohort of 39 functional and 23 silent corticotroph adenoma indicated that there was no difference regarding the age of the participants, as well as hormonal profile, size, and invasiveness of the tumor between patients with and without *USP8* variants. However, *USP8*-mutated adenomas showed significantly higher SSRT5 expression compared to the wild-type ones [26].

In a cohort study, *USP8*-mutated FCA patients were dominantly women and showed lower ACTH levels and smaller tumor size, but no difference in cortisol level. Remission rate was significantly higher in *USP8*-mutated patients compared to the wild-type ones. Moreover, *USP8*-mutated adenomas were more likely to express SSTR5 [30]. Genetic analysis of 51 FFPE tumors (21 *USP8*-mutated and 30 wild-type) indicated significantly higher SSTR5 immunoreactivity score in USP8-mutated tumors, regardless of mutation type. Moreover, in vitro study of 24 corticotroph tumors freshly obtained after TSS indicated a significantly better response to Pasireotide treatment, defined as suppression of ACTH secretion, in human corticotroph tumors carrying *USP8* variants [31].

A more recent study aimed to investigate the impact of *USP8* variants on in vitro response to Pasirotide in primary cultures obtained from 7 FCAs and also in murine corticotroph tumor cells. *USP8* variant in both primary cultured cells and AtT20 cells was associated with higher SSTR5 expression. Moreover, this study indicated although associated with SSTR5 upregulation, mutations at the amino acid 718 of *USP8* are not associated with a favorable response to pasireotide, whereas *USP8* variants at the amino acid 720 might preserve pasireotide responsiveness [32].

Inhibition of EGFR using Gefitinib, a tyrosine kinase inhibitor, in surgically resected human and canine corticotroph cultured tumors suppressed expression of POMC. Moreover, Blocking EGFR activity in mice attenuated POMC expression, inhibited corticotroph tumor cell proliferation, and induced apoptosis [33]. Araki et al. conducted a study to investigate the utility of EGFR as a therapeutic target for CD. EGFR expression was observed by 2.5 months in transgenic (Tg) mice; and aggressive ACTH-secreting pituitary adenomas with features of Crooke’s cells developed by 8 months with 65% penetrance observed. Moreover, they used the EGFR tyrosine kinase inhibitor Gefitinib to confirm reversibility of EGFR effects on ACTH. Gefitinib suppressed tumor POMC expression and downstream EGFR tumor signaling. Plasma ACTH level and pituitary tumor size was significantly lower in Gefitinib group [34].

Another experimental study investigated the effect of Lapatinib, a potent tyrosine kinase inhibitor, on ACTH production and cell proliferation in AtT-20 mouse corticotroph tumor cells. Lapatinib inhibits EGFR. In this study, Lapatinib decreased proopiomelanocortin (POMC) mRNA levels and ACTH levels in AtT-20 cells and also inhibited cell proliferation and induced apoptosis. Inhibition of EGFR signaling contributes to the inhibition of ACTH production and cell proliferation in corticotroph adenomas [35].

The effect of a potent and selective Jak2 inhibitor, SD1029, on ACTH production and proliferation investigated in mouse AtT20 corticotroph tumor cells. They observed that Jak2 inhibitor SD1029 decreased both POMC transcript levels and basal ACTH levels. These in vitro experiments suggest the Jak2 inhibitor suppresses both the autonomic synthesis and release of ACTH in corticotroph tumor cells. SD1029 was also found to inhibit AtT20-cell proliferation. In addition, SD1029 decreased and increased PTTG1 and GADD45β transcript levels, respectively. They seem to contribute, in part, in the Jak2-induced suppression of cell proliferation and ACTH synthesis [36]. An experimental study examined the effect of USP8 inhibitor on EGFR expression level, and cell viability using AtT20 cells treated with 9-ehtyloxyimino9H-indeno (1, 2-b) pyrazine-2,3-dicarbonitrile, a synthesized USP8 inhibitor. This study demonstrated that treatment with USP8 inhibitor, 9‑ehtyloxyimino9H‑indeno(1,2‑b) pyrazine‑2,3 dicarbonitrile, suppresses ACTH secretion, cell viability, and promotes cell apoptosis in AtT20 cells suggesting that USP8 inhibitor could be a new therapeutic candidate for CD [37].

Kageyama et al. investigated the effects of a potent USP8 inhibitor, DUBs-IN-2, on ACTH production and cell proliferation in mouse corticotroph tumor (AtT-20) cells. DUBs-IN-2 decreased Proopiomelanocortin (POMC) mRNA and ACTH levels. Furthermore, DUBs-IN-2 decreased At-20 cell proliferation and induced apoptosis in corticotroph tumor cells [38]. Another study explored the potential effect of the USP8 inhibitor RA-9 on USP8-WT human tumor corticotroph cells and murine AtT-20 cells. RA-9 significantly decreased cell proliferation and increased cell apoptosis in AtT-20 cells. Moreover, RA-9 reduced ACTH release by USP8-mutant cells. The combined treatment with RA-9 and pasireotide resulted in more efficient in inhibiting ACTH secretion compared with RA-9 or pasireotide alone. Furthermore, similar to pasireotide, RA-9 was able to significantly reduce phospho- ERK1/2 levels in both AtT-20 cells and primary cultured cells from corticotropinomas [39].

Another study, investigating the *USP8* variants and genes involved in cell cycle regulation, looked for the role of *USP8* variants or a changed p27 level in the response to Palbociclib, Flavopiridol, and Roscovitine, in vitro, using murine corticotroph AtT-20/D16v-F2 cells. They did not found any significant difference in cell viability or cell proliferation between the AtT-20/D16v-F2 cells overexpressing wild-type and mutated *USP8* that were treated with cell cycle inhibitors. There was also no difference in the response to inhibitors of CKDs in the cells with overexpression of p27 and control cells [17].

## Analytical conclusion

In our series, we found two *USP8* variants including a 7-bp deletion variant, c.2151_2157delCTCCTCC, p. Ser718GlnfsTer3, and a missense variant, c.2159 C > G, p. Pro720Arg (rs672601311) in exon 14. Moreover, the systematic review of the published data indicated that 35% of corticotroph adenomas harbor *USP8* variant the most of which was found in the 720 code region, p. Pro720Arg. Similar to the most previous studies, the *USP-8* mutated patients were women, presented with micro-adenoma and experienced recurrence after initial remission.

We systematically reviewed the literature regarding the *USP8* variant in corticotroph adenomas and classified the results into three categories; including *USP8* variants and the related pathways, genotype-phenotype correlation in *USP8*-mutated individuals, and *USP8* and EGFR as potential therapeutic targets.

Different *USP8* variants are identified in corticotroph adenomas. Activation of the EGFR pathway is a well-established consequence of *USP8* variants [6, 15]. But there is inconsistency regarding the role of *USP8* variants in cell cycle regulation in corticotroph adenomas. Some studies showed no difference in the gene expression of the cell cycle regulators CDKN1B (P27), CCNE1 (CYCLIN-E1), CCND1 (CYCLIN-D1), CDK2, CDK4, and CDK6 according to *USP8* variant status [21]; while the others indicated *USP8*-mutated tumors have lower CDKN1B, CDK6, CCND2 and higher CDC25A expression [20]. Moreover, demethylation of the first promoter is affected with *USP8* variant status [19]. However, more studies are required to establish the pathway underlying the *USP8* variants.

Data regarding sex, age, hormonal level, tumor size, and clinical outcomes in *USP8*-mutated individuals with FCA are relatively consistent among different studies. The *USP8* variant seems to be associated with younger age and is more likely to occur in women. Meta-analysis of data from ten series indicated *USP8* variant is 2.63 times higher in women than in men [40]. Since CD is more prevalent in young women, the potential effect of estrogen on the growth of *USP8*-mutant corticotroph cells has been hypothesized. There is evidence that corticotroph cells express estrogen receptors [41]. Moreover, in vitro studies indicated estrogen can stimulate corticotroph cell proliferation mediated by EGFR signaling pathways [42]. More precise studies are required to better explain the age-sex distribution of *USP8* variant in patients with CD.

Results regarding the hormonal pattern among the series are partly controversial. Two series indicated significantly higher levels of ACTH and UFC in *USP8*-mutated patients compared to the wild-type ones [7, 24]. Moreover, one study demonstrated the expression levels of EGFR were positively correlated with ACTH and cortisol levels [10]. Conversely, one study showed a significantly lower ACTH level in patients with the *USP8* variant [30]. However, in a systematic analysis of the two series the correlation of UFC and *USP8* variant did not reach a significant difference, this might be due to the small number of cases included in the analysis [40].

There are also some discrepancies on tumor size and invasiveness in *USP8*-mutated tumors. Some studies indicated a significant smaller size in *USP8*-mutated tumors, while others showed a significant larger size in *USP8*-mutated tumors. But some study found no significant difference regarding tumor size and invasiveness between *USP8*-mutated and wild-type tumors. A recent systematic analysis of magnetic resonance imaging (MRI) findings from individuals with CD indicated *USP8*-mutated tumors are more likely to be less than 10 mm compared to wild-type ones [40]. Moreover, a cohort of 60 patients with FCA indicated smaller tumor size and less invasiveness in *USP8*-mutated tumors [30]. In contrast to these findings, a cohort of Brazilian patients observed a tendency toward more somatic *USP8* variant in tumors more than 10 mm in size [40]. These discrepancies might be due to the different methods used for extraction of MRI data.

Considering the clinical outcomes, most studies indicated a higher remission rate except for one that showed a significantly lower rate of remission in *USP8*-mutated patients [22, 25, 28, 30]. Moreover, some studies demonstrated a higher rate of recurrence in carriers of *USP8* variant [24, 27, 42]. However, other studies found no significant difference neither in the initial remission nor in the late recurrence rate between the carriers of *USP8* variant and the individuals with wild-type *USP8.* The inconsistency in the results might be due to the lack of a systematic protocol for evaluation of these patients. Moreover, the number of patients included in the different studies was relatively low. Further multicenter prospective studies with the same protocol are required to yield more consistent information regarding the influence of *USP8* variant on the clinical presentation as well as early and late outcomes of FCAs.

There are promising studies regarding *USP8*-targeted therapy. We found evidence that *USP8-*mutated tumors have higher SSRT5 expression [30, 31]. Moreover, in vitro studies demonstrated that Pasirotide suppressed ACTH secretion significantly more in the *USP8*-mutated tumors than in wild-type ones [31]. These evidences suggest that *USP8* mutational status could be used as a marker of Pasirotide response in CD. Furthermore, *USP8*-mutated tumors are more likely to express EGFRs compared to the wild-type ones [6]. Inhibition of EGFR using Gefitinib and Lapatinib has been associated with promising results regarding the EGFR-targeting therapy in CD [33–35]. Moreover, experimental studies of two *USP8* inhibitors, 9‑ehtyloxyimino9H‑indeno (1,2‑b) pyrazine‑2,3 dicarbonitrile and DUBs-IN-2, have shown their potential to suppress POMC expression and ACTH secretion, decrease cell proliferation, and promote apoptosis [37, 38].

In summary, the studies investigated the association of *USP8*- variants and clinical manifestations as well as clinical outcomes of the corticotroph adenomas are partly inconsistent. More precise multicenter studies are required to yield more consistent information regarding the phenotype-genotype correlation and to develop effective targeted therapies.

### Electronic supplementary material

Below is the link to the electronic supplementary material.

Supplementary Material 1

## Data Availability

The datasets used and/or analyzed during the current atudy are available from the corresponding author on reasonable request.

## Abbreviations

ABI: Applied Biosystems
ACTH: Adrenocorticotropic Hormone
CCA: Crooke Cell Adenomas
CD: Cushing’s Disease
DNA: Deoxyribonucleic Acid
EGFR: Epidermal Growth Factor Receptor
Erk: Extracellular Signal-Regulated Kinases
FCAs: Functional Corticotroph Adenomas
FCPA: Functional Corticotroph Pituitary Adenoma
FFPE: Formalin-Fixed And Paraffin-Embedded
ICH-GCP: International Conference On Harmonization Of Good Clinical Practice
IHC: Immunohistochemistry
MRI: Magnetic Resonance Imaging
PCR: Polymerase Chain Reaction
PRISMA: Preferred Reporting Items For Systematic Reviews And Meta-Analyses
RNA-seq: RNA Sequencing
SCA: Silent Corticotroph Adenomas
TSS: Transsphenoidal Surgery
USP8: Ubiquitin-Specific Peptidase
USP48: Ubiquitin Specific Peptidase 48
