# Supplementary material for: Targeted analysis of Ubiquitin-Specific Peptidase (USP8) in a population of Iranian people with Cushing’s disease and a systematic review of the literature
Source: BMC Endocr Disord. 2024 Jun 11;24:86. doi: 10.1186/s12902-024-01619-z (PMC11165846; doi:10.1186/s12902-024-01619-z)
Supplement: Supplementary file 1 — Supplementary Material 1 [file 12902_2024_1619_MOESM1_ESM.docx]

Supplementary Table 1. Baseline characteristics of the participants and the clinical outcomes after surgery.

| patient | sex | Age(yrs) | Cortisol  8am  (mic/dl) | ACTH  (pg/ml) | UFC  (mic/24h) | Tumor size  (mm) | Outcome after surgery |
| --- | --- | --- | --- | --- | --- | --- | --- |
| Number 1 | F | 45 | 22 | 164 | 1153 | 9 | cure |
| Number 2 | F | 46 | 25 | 79 | 316 | 6 | cure |
| Number 3 | F | 59 | 26 | 140 | 922 | 9 | cure |
| Number 4 | F | 36 | 20 | 92 | 400 | 5 | cure |
| Number 5 | F | 42 | 24 | 67 | 670 | 6 | cure |
| Number 6 | F | 46 | 30 | 110 | 870 | 7 | cure |
| Number 7 | F | 43 | 22 | 104 | 550 | 7 | cure |
| Number 8 | F | 37 | 32 | 140 | 980 | 9 | cure |
| Number 9 | F | 44 | 28 | 98 | 670 | 5-3 | cure |
| Number 10 | F | 22 | 29 | 34 | 504 | 11 | persistent |
| * Number 11 | F | 42 | 27 | 44 | 536 | 5.5 | Recurrence after initial remission |
| * Number 12 | F | 38 | 24 | 47 | 580 | 6 | Recurrence after initial remission |
| Number 13 | M | 40 | 43 | 162 | 1100 | 9 | Recurrence after initial remission |
| Number 14 | M | 17 | 43 | 113 | 750 | 6 | cure |
| Number 15 | M | 47 | 36 | 112 | 980 | 9 | cure |
| Number 16 | M | 65 | 22 | 84 | 550 | 12 | persistent |
| Number 17 | M | 45 | 27 | 87 | 670 | 9 | cure |
| Number 18 | M | 39 | 23 | 45 | 439 | 8 | cure |
| Number 19 | M | 34 | 25 | 44 | 370 | 7 | cure |

ACTH; Adrenocorticotropic hormone, UFC; urine free cortisol. *Patients number 11 and 12 showed USP8 mutation.
